# Effects, Side Effects and Contraindications of Relaxation Massage during Pregnancy: A Systematic Review of Randomized Controlled Trials

**DOI:** 10.3390/jcm10163485

**Published:** 2021-08-06

**Authors:** Stephanie M. Mueller, Martin Grunwald

**Affiliations:** Haptic Research Lab, Paul-Flechsig-Institute of Brain Research, University of Leipzig, Liebigstrasse 19 Haus C, 04103 Leipzig, Germany; mgrun@medizin.uni-leipzig.de

**Keywords:** antenatal massage, pain, stress, depression, anxiety

## Abstract

Healthcare professionals and expecting mothers frequently voice concerns that massages during pregnancy might cause complications or premature labor. This PRISMA review outlines current results on effects, side effects and contraindications of relaxation massage during pregnancy. Inclusion criteria: all randomized controlled trials (RCT) comparing relaxation massage during pregnancy with standard care or standard care plus another intervention (i.e., progressive muscle relaxation). Restrictions were full text availability and English language. Results: 12 RCT were included. Trials had good methodological quality but unknown risk of bias. All women were at least 12 weeks gestation at the start of the study. The main benefits of massage during pregnancy were: reduced stress, back and leg pain, depression and anxiety; increased immune response; increased serotonin and dopamine levels; higher fetal birth weight and reduced risk of preterm delivery. Only 2 RCT reported potential side effects of massage, which were minor and transient. Seven RCT excluded women with difficult pregnancies or preexisting complications, five studies did not report preexisting conditions. Those obstetric or postnatal complications that occurred were most likely unrelated to massage treatments. In healthy pregnant women without complications, relaxation massage has positive effects throughout pregnancy. Precautions for massage during pregnancy (i.e., to prevent pulmonary embolism) are discussed.

## 1. Introduction

Massage treatments are a potent tool to alleviate stress, anxiety, pain and depression in various patient groups [1,2,3,4,5]. Psychosocial stress and depression are among the major risk factors during pregnancy, which may negatively impact the mother’s health as well as the development and growth of the fetus [6]. Untreated or insufficiently treated prenatal depression is linked to complications during pregnancy [7,8], such as preeclampsia [9], early labor [10], and more frequent caesarian deliveries [11,12]. Aside from that, newborns delivered by mothers suffering from prenatal depression are more often in need of intensive care: children born by depressed mothers have a lower birth weight and a smaller head circumference than children delivered by healthy mothers [7]. In addition, dyspnea and/or feeding disorders are more prevalent [12]. Depression during pregnancy can also lead to growth retardation and can have a negative impact on cognitive, motoric and emotional developments during infancy and childhood [13,14,15,16]. Furthermore, prenatal depression is one of the leading risk factors for postpartum depression which, in turn, bears significant developmental risks during infancy and early childhood [17]. 

Unfortunately, treatment of prenatal depression is complicated by the fact that the use of selective serotonin reuptake inhibitors (SSRI) during pregnancy is in itself suspected of negatively influencing fetal development and contributing to complications during pregnancy [12,18,19,20,21]. For this reason, it is imperative that further research investigates non-pharmacological treatments in support of psychotherapeutic approaches [8].

Aside from alleviating psychological factors (anxiety, stress, depression), many women seek massage treatments to reduce low back and/or pelvic girdle pain during pregnancy [22,23].

However, some healthcare professionals still do not treat pregnant women out of fear of inducing premature contractions or causing physical harm, and expecting mothers voice concerns that massages during pregnancy might be unsafe or cause complications [24,25]. Recent systematic reviews with meta-analyses have investigated the effects of massage and other complementary therapies on pregnant women’s anxiety and depression [26,27]. The authors found promising effects. However, so far, no systematic review has analyzed which adverse side effects or complications may be caused by massage during pregnancy. Furthermore, no systematic review has summarized all currently known effects that may be achieved through regular massage during pregnancy. The present review will fill that gap by summarizing which positive effects and which adverse side effects or complications on mother and child have been reported in randomized controlled trials. Additionally, we will review how potential contraindications were handled and which precautions should be taken. 

## 2. Methods

Literature searches were conducted with the following major electronic databases from their inception to June 2021: The Cochrane Central Register of Controlled Trials, PubMed, Medline, and Web of Science.

Key search terms were: “massage” AND “pregnancy” AND “relaxation” OR “safety” OR “side effects” OR “pain” OR “contraindications”.

As inclusion criteria, we required that the study samples were humans; hands on relaxation massages were performed during pregnancy; the articles were full texts and available in English language, and that the study designs were randomized controlled trials (RCT). Exclusion criteria: We excluded conference abstracts, case reports, studies without a control group and articles without full texts. Furthermore, all studies were excluded that analyzed massage during labor or perineal massage. This review focusses on relaxation massage and does not investigate acupuncture, reflexology, manual therapy, osteopathic techniques, electrical stimulation or other complementary pain relief techniques like water injection or hot/cold compresses. 

The methodological quality of each included study was assessed based on the tool by National Institute of Health [28] (see Table 1). Possible answers to the nine items were affirmative (+), negative (–), or other, including “cannot determine”, “not applicable”, and “not reported”, which were considered unclear (?) answers. Sum scores were calculated to classify the quality of the studies as good (7–9), fair (4–6), or poor (≤3). 

Risk of bias assessment was based on Cochrane Bias Methods Group [29] assessment tool (Table 1). Possible answers to the five bias categories were high risk of bias (+), low risk of bias (–), or other, including “cannot determine”, “not applicable”, and “not reported”, which were considered unclear (?) risk of bias. The study quality and risk of bias were determined by comparing rating agreement, with consensus required between the two raters. Discrepancies in study quality rating were reconciled via discussion of the individual items.

## 3. Results and Discussion

**Figure 1:**
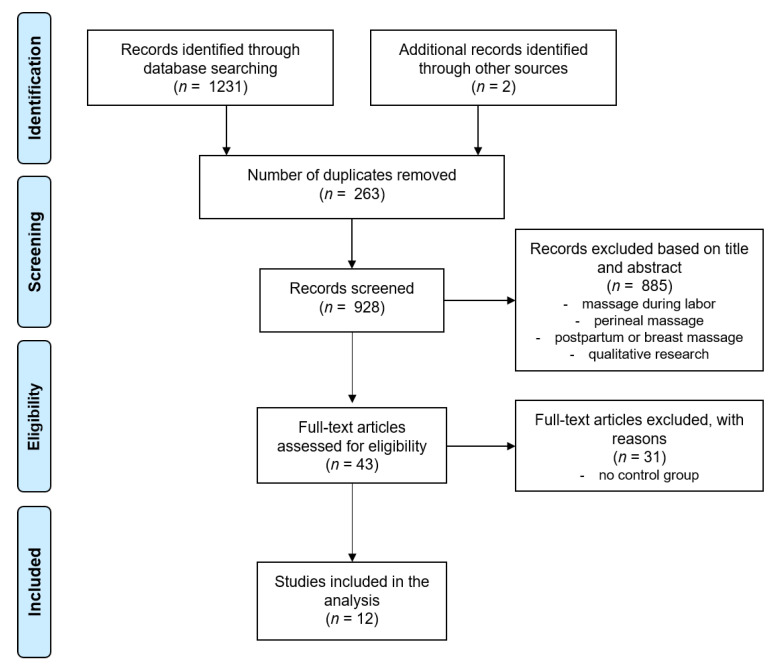
PRISMA flow chart of the searching and selection process.

By the initial search 1231 potential articles were identified. Of these records, 885 were excluded because they were unrelated to the research topic based on their title or abstract. After all duplicates and trials without a control group were excluded, 10 randomized controlled trials (RCT) remained. By screening the reference lists of relevant articles, two additional studies were identified. Finally, 12 RCT were found to be eligible and included in this review. The PRISMA flow chart is presented in Figure 1. The characteristics of all included studies are described in Table 2.

### 3.1. Effects of Massage during Pregnancy on Mood, Sleep, Pain and Other Pregnancy-Related Discomforts

Unless otherwise indicated, massages used in the reported studies lasted approximately 20 min, were performed a total of 5 to 10 times (1–2 per week), between the 16th and 36th week of pregnancy. The women receiving treatments were laying on one side with a pillow in their backs and between their legs, and were massaged on the head, shoulders, back, arms, hands and feet. After 10 min, the women turned to the other side and the treatment was repeated (for detailed description, see [30]). In order for the massage to be effective, moderate pressure should be applied, which the women perceive as pleasant and non-painful [44]. Massage treatments that are either too painful or too gentle have been reported to cause an activation of the autonomous nervous system [44,45], which may prevent the relaxing and stress-reducing effect.

To date, five RCT have outlined the effects of massages on psychologically and physically healthy pregnant women with uncomplicated pregnancies [31,32,33,39,40]. In the first study, the women received 20 min massage treatments twice a week for a total of five weeks [31]. The effects were compared with a control group that was instructed to perform progressive muscle relaxation twice a week. Both groups reported a decline in leg pain and anxiety and showed significantly higher dopamine levels at the end of the study. For all other dependent variables, only those women who received massage treatments reported significant improvements:

- back pain was reduced;
- their mood improved;
- they reported calmer and deeper sleep;
- noradrenaline levels were reduced.

**Figure 2:**
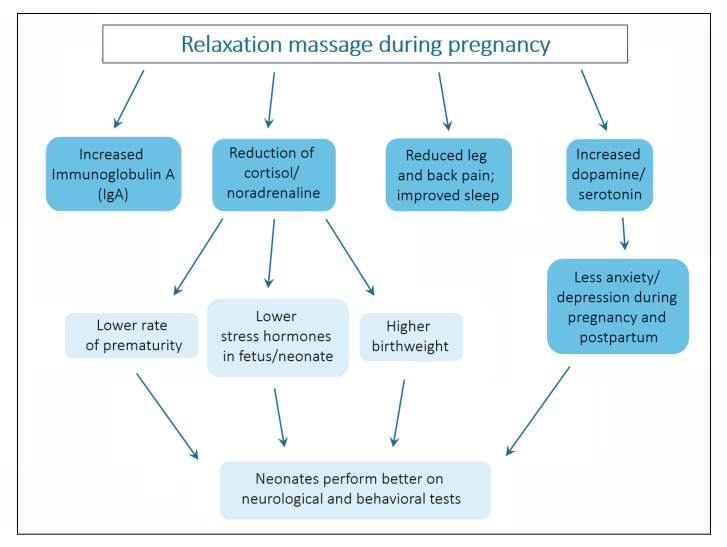
Effects of massage during pregnancy on mother (dark blue) and fetus (light blue).

The other studies support and complement these findings (for a summary, see Figure 2). In one study, 150 pregnant women were randomized into a massage and a standard care control group [33]. The authors found statistically significant differences in favor of the massage group regarding headache, backache, muscle cramp, sleep disturbance and anxiety, while there was no difference related to joint pain.

Recent meta-analyses have shown that manual therapy techniques (craniosacral therapy, osteopathic manipulative treatment, chiropractic) are effective treatment tools for pregnancy-related low back pain and pelvic girdle pain when compared to standard care control groups [22,23]. However, compared to sham ultrasound, there was no difference in pain reduction through osteopathic manipulative treatment. In line with the above reported positive effects on pregnancy-related pain through regular relaxation massage, these results indicate that intense manual techniques may not be necessary to reduce headaches, back pain and muscle cramps during pregnancy. Moderate touch interventions may be enough to generate significant relieve from pregnancy-related discomforts.

One study focused on massage effects on pregnancy-related non-clinical anxiety [32]. They found significant improvements in women receiving massage treatments and women performing guided imagery exercises, while anxiety scores in the standard care control group increased. All participating women were primiparous, healthy and had not experienced any stressful life events during the past 6 months. Interestingly, women with lower education levels were less persistent in performing the guided imagery exercises. Massage treatments, on the other hand, were highly accepted independent of education level. 

Within the scope of the fourth study, the cortisol and immunoglobulin A-levels (IgA) of women who had received massage treatments were compared with women who had received standard medical care [40]. The women in the intervention group were massaged for 70 min every second week (with aroma oils, ten times total) between the 16th and 36th week of pregnancy. Immediately after each massage treatment, significantly reduced cortisol and increased IgA levels were measured. In the intervention group, pre-massage IgA levels rose continuously across the 10 measurements, indicating that the regularly administered massage treatments had a positive cumulative effect on the immune system [40].

A fifth study compared the effects of partner-delivered relaxation massage and self-directed stress-management training on subclinical psychological symptoms [39]. The participants filled out online questionnaires to report their perceived depression, anxiety and stress. The authors found that symptoms decreased in both groups, with no significant difference between the two groups. 

These and other studies indicate that the desired effects can be achieved through massages administered by the partner [14,46] or by a professional massage therapist/physical therapist [34]. Massage treatments performed by the partner may have additional positive impact on the quality of the relationship. As research shows, partner massages have the potential to both alleviate existing stress and to prevent psychosocial stress by reducing the number of relationship conflicts [8,35].

### 3.2. Effects of Massage during Pregnancy on Women with Prenatal Depression

Seven RCT investigated the effects of massage on clinically depressed pregnant women [14,30,34,35,36,37,38]. These studies confirm the benefits that were observed in healthy pregnant women. Depressed pregnant women who received massage treatments reported lower depression and anxiety levels [34,35,36,37] as well as less leg and back pain [34,35] than those who did not receive relaxation massages. In one study, pregnant women who received group psychotherapy plus massage treatments attended more psychotherapy sessions over the course of several weeks than women who received only psychotherapy [38]. The experimental group, which received both massage treatments and psychotherapy, demonstrated a larger decline in depression, anxiety and cortisol levels than the control group treated solely with psychotherapy [38]. Further studies have confirmed the positive effect of massage on the cortisol [30,47] and noradrenaline [30] concentrations of depressed pregnant women. In addition, the concentrations of dopamine and serotonin have been found to increase significantly after massages, a fact that underpins the reports of decreased anxiety and depression levels [30]. Furthermore, two studies compared the Hamilton Depression Rating Scale scores (HDRS) of pregnant women who received either 8 weeks of acupuncture or 8 weeks of massage therapy [36,37]. They found significant reductions of HDRS in both groups that were similar to treatments with antidepressants or cognitive therapy (reduction of HDRS approximately from 21 to 14) [36,37]. Remission rates were 34.8% in the acupuncture group and 31.2% in the massage group [37]. Moreover, only 2% of women in the massage group reported side effects (tiredness after treatment), while almost half of participants in the acupuncture group experienced mild or transient side effects (e.g., nausea, headache or sleep disturbance after treatment). In line with these results, a current meta-analysis of four RCT (all four conducted by Field et al.) revealed a moderate effect of massage therapy on pregnant women’s depressive symptoms [26]. 

### 3.3. Effects of Massage during Pregnancy on Fetal Development

Regular relaxation massages during pregnancy may have positive effects on the development and growth of the fetus. So far, these effects have been primarily investigated in depressed pregnant women. However, similar effects can be expected in association with other psychosocial stress factors. The four included studies that investigated fetal outcome showed that depressed women who received regular relaxation massages during pregnancy had lower incidence of premature births and delivered children with higher birth weight than women in the control groups [14,30,31,34]. Similar effects were achieved through 20 min yoga sessions once or twice a week [14,34]. Furthermore, the newborns of depressed mothers who received massages had lower cortisol levels and scored better on behavioral and neurological tests than neonates of depressed mothers who had not received massages [14]. Also, massage during pregnancy may be associated with fewer neonatal complications in the infants [31]. 

In a further study without a control group, 64 neonates (mean age 6.8 days) of depressed mothers who received massage therapy during pregnancy were assessed on their behaviors during 15 min observations and on their performance on the Brazelton Neonatal Behavior Assessment Scale [48]. Half of the mothers had received moderate pressure massage and the other half received light pressure massage. The results suggested that moderate pressure might have more positive effects on neonatal outcomes than light pressure. Neonates whose mothers had received moderate pressure massage spent a greater percent of the observation time smiling and vocalizing. On the Brazelton scale, they reached better scores on the orientation, motor, excitability, and depression clusters [48]. 

To the best of our knowledge, to date, only one research group has analyzed the effects of massage during pregnancy on neonatal outcomes. To increase reliability, replication studies are needed. 

### 3.4. Side Effects, Complications, Precautions and Contraindications 

Only two of the randomized controlled studies reported potential side effects of massage or investigated contraindication for massage during pregnancy [37,39]. As a precaution, seven of the RCT excluded women with difficult pregnancies or preexisting physical complications (i.e., placenta previa, premature labor, blood clotting disorders or diabetes) [14,32,33,34,35,39,40]. The remaining five studies did not report whether the women had any known preexisting conditions (see Table 2).

In women with uncomplicated pregnancies, only minor transient side effects (tiredness) were reported that were likely caused by the massage [37,39]. Eight studies did not report if obstetric or postnatal complications occurred (see Table 2). Two studies reported fewer obstetric complications in the massage group than in the control group [30,31], and one study reported fewer neonatal complications in the massage group [31]. One study that did not explicitly exclude women with complicated pregnancies reported several complications/adverse events during the course of the study [37]. The study investigators and the Data Safety and Monitoring Board of that trial rated all complications and events as unrelated to the massage treatment [37]. 

These results indicate that healthy women with uncomplicated pregnancies may receive massages during the entire course of the pregnancy. The results are further supported by an investigation with pre/post design but no control group, that explicitly investigated side effects after a single session of full-body Swedish massage in expecting mothers (*n* = 101) with a one-week follow-up [49]. The authors reported that 40% of participants experienced one or more of the following side effects: post-treatment muscle soreness, headache, exacerbation of musculoskeletal symptoms, tiredness after treatment and dizziness. Obstetric complications did not occur: no mother or child incurred physical harm from the massage treatments.

Some practitioners believe that stimulation of certain acupressure points or reflexology points during pregnancy may induce labor. Even if massage therapists are unfamiliar with these techniques, they may refrain from massaging pregnant women to prevent unwillingly touching these points. However, there are no scientific indications to support that claim. In a randomized controlled study, no effects on the onset of labor could be determined as a result of acupressure within 97 h [50]. In women who received foot reflexology treatments during pregnancy, no adverse side effects have been reported in studies [51,52,53]. 

The main criterion is what benefits the pregnant woman and what she perceives to be pleasant or non-painful. However, since conditions can change, health status should be inquired before each treatment and, when in doubt, in consultation with the obstetrician. In order to prevent adverse side effects, it is advisable to consult with the obstetrician for women with complicated or high-risk pregnancies and decide, on a case-by-case basis, which treatments may be save.

To date, only one study (not randomized, no control group) seems to exist that provided hospitalized women with high-risk pregnancies with various touch interventions (including massage, healing touch and reflexology) during their antepartum hospital stay [54]. No adverse reactions occurred in any of these women. This means, however, that there is still little evidence-based knowledge on whether massage would ameliorate or worsen these conditions.

In any case, some precautions for massage during pregnancy should be taken to prevent adverse side effects:

#### 3.4.1. Risk of Thrombosis

Pulmonary embolism as a complication of deep venous thrombosis is the leading cause of death during pregnancy in developed countries [55]. Women are 5 times more likely to develop deep venous thrombosis (DVT) when pregnant [55]. DVT can be difficult to diagnose, as its symptoms can be very similar to discomforts that accompany normal pregnancies, like swelling of the legs and back pain. Leg massage during pregnancy with unrecognized DVT can be life threatening [56]. Therefore, no massages should be performed on the deep muscle tissue of the arms and especially not the legs, in order to avoid loosening blood clots or causing hematomas. However, superficial lymphatic drainage and gentle stroking touches to alleviate edemas are allowed [56]. 

#### 3.4.2. Lateral Position

Massages should be performed in lateral position if the woman is laying down; sitting positions are also possible. Pregnant women should not be massaged while lying on their stomachs, since it is uncomfortable for many expecting mothers and may hinder relaxation. In order to prevent the vena cava syndrome, pregnant women should not remain in a supine position for an extended period of time [57].

#### 3.4.3. Abdominal Massage

During pregnancy, women should not be massaged on the abdomen, as it can lead to placental or uterine rupture and may cause pregnancy loss and even maternal death [58].

#### 3.4.4. Essential Oils

Since some essential oils are suspected of inducing contractions, no highly concentrated essential oils (aromatherapy) should be applied during pregnancy. Massages with diluted essential oils (such as 2% lavender oil [40]) have not been shown to lead to adverse side effects. During the birth process, the pain-relieving and relaxing effects of essential oils (inhalation and massage) may prove helpful [59,60].

## 4. Conclusions

During pregnancy, the main concerns are aggravating pregnancy complications and causing thrombosis. Therefore, women with difficult pregnancies or preexisting complications should only be massaged upon consultation with the women’s OBGYN. Generally, deep tissue massage should be avoided to prevent detaching blood clots. Apart from these precautions, regular relaxation massages can be used throughout pregnancy to diminish stress and various pregnancy-related discomforts. Massages during pregnancy have been shown to reduce symptoms of depression and anxiety, attenuate leg and back pain, reduce cortisol levels, and show positive effects on immune function. Massage treatments show more positive effects than other relaxation techniques like progressive muscle relaxation. Effects on prenatal depression are comparable to psychotherapy and antidepressants.

So far, only few systematic studies exist that investigate the effects of relaxation massages during pregnancy and their potential impact on mother and child. A limited number of researchers conducted the existing studies. Especially, possible positive effects on prematurity, fetal growth and pregnancy complications need further investigation. 

Presently, the therapeutic potential of massage treatments is often underestimated and not optimally utilized. It is necessary to rethink the biological and psychological effects of massage treatments and to make more use of this potent tool. 

## Figures and Tables

**Table 1:** Methodological quality and risk of bias of included studies.

Reference	Methodological Quality										Risk of Bias				
	1	2	3	4	5	6	7	8	9	Quality Rating ^a^	A	B	C	D	E
[14]	+	+	?	+	+	+	+	+	+	8	?	?	?	?	?
[30]	+	+	?	?	+	?	+	+	+	6	?	?	?	?	?
[31]	+	−	−	?	+	?	+	+	+	5	?	?	?	?	?
[32]	+	?	+	+	+	+	+	?	+	7	−	?	?	+	?
[33]	+	?	?	+	+	+	?	+	?	5	?	?	?	+	?
[34]	+	+	?	−	+	+	+	+	+	7	?	?	?	−	?
[35]	+	−	−	+	+	+	?	+	+	6	?	?	?	?	?
[36]	+	+	−								?	?	−	+	?
[37]	+	+	−	+	+	+	+	+	−	7	?	?	−	+	?
[38]	+	+	?	?	+	+	+	+	−	6	?	?	?	+	?
[39]	+	+	−	+	+	+	−	+	+	7	?	?	?	?	?
[40]	+	+	−	+	+	+	+	+	−	7	?	?	?	?	?

Affirmative/high risk of bias (+), negative/low risk of bias (−), or other, including “cannot determine”, “not applicable”, and “not reported”, which were considered unclear (?) answers. Methodological quality assessment based on [28]: 1 = Was the study question or objective clearly stated?; 2 = Was the study population clearly and fully described, including a case definition?; 3 = Were the cases consecutive?; 4 = Were the subjects comparable?; 5 = Was the intervention clearly described?; 6 = Were the outcome measures clearly defined, valid, reliable, and implemented consistently across all study participants?; 7 = Was the length of follow-up adequate?; 8 = Were the statistical methods well described?; and 9 = Were the results well described? ^a^ Quality rating was good (7–9), fair (4–6), or poor (≤3). Risk of bias assessment was based on [29]: A = Selection bias (randomization protocol and allocation concealment), B = Performance bias (blinding of participants/personnel), C = Detection bias (blinding of outcome assessment), D = Attrition bias (incomplete outcome data), E = Reporting bias (selective outcome reporting).

**Table 2:** Description of included studies.

Reference	Sample Size Mean Age/Ethnicity	GA in Weeks at Study Start	Groups and Interventions	Assessment Tools (Anxiety, Depression, Pain, etc.)	Outcome Mothers	Outcome Neonates	Side Effects	Complications	**Precautions**
[14]	200 depressed women were recruited and randomized, 149 took part (61 control, 88 intervention); Mean (SD) age: control group: 25.2 (5.8); massage group: 27.2 (6.5); USA: 57% Hispanic, 38% African American, and 5% Non-Hispanic	Between 16 and 20 weeks gestation	Two groups: massage group received 2 moderate pressure massages per week for 12 weeks by their significant other Control: standard care	Mother: SCID; CES-D; STAI; The Daily Hassles Scale [41]; Sleep Disturbance Scale [42]; back pain VAS Neonate: Saliva cortisol; birthweight; gestational age; Brazelton Neonatal Behavior Assessment Scale (BNBAS).	Long-term effects (first vs. last day of study): less depression and back pain in massage group; No diff. between groups in anxiety, sleep disturbance or daily hassles	Massage group: lower incidence of prematurity and low birthweight; neonates: lower cortisol levels; better performance on BNBAS habituation, orientation and motor scales	Not assessed	Not assessed	Only low-risk, uncomplicated pregnancies, healthy mothers
[30]	84 **depressed** pregnant women were recruited plus 28 non-depressed women; Mean (SD) age: 28.8 (5.7); USA: 46% Caucasian, 39% Hispanic, 12% African American and 3% Asian	Between 18 and 24 weeks gestation (M = 22.9)	Four groups: massage therapy group by significant other; progressive muscle relaxation group; 20 min sessions per week for 16 weeks; two control groups (depressed/ non-depressed) standard prenatal care	STAI; Profile of Mood States Scale (POMS); CDC-D; pain VAS; Urine: cortisol, catecholamines (norepinephrine, epinephrine, dopamine) and serotonin (5-HIAA) Postnatal: Obstetric Complications (OCS) and Postnatal Factor (PNF) Scales; Brazelton Neonatal Behavior Assessment Scale (BNBAS)	Immediate effects (pre/post session): Massage: lower levels of anxiety and depressed mood, less leg and back pain. PMR: less leg pain Control groups: no change Long-term effects (first vs. last day of study): Massage: higher dopamine and serotonin levels, lower levels of cortisol and norepinephrine PMR and control groups: no change	Massage group: lower incidence of prematurity and low birthweight; better performance than depressed control on BNBAS: habituation, range of state, autonomic stability, withdrawal scales, depressed scale, motor maturity	Not assessed	Massage group had less obstetric complication than PMR or depressed control	Not reported
[31]	26 pregnant women; mean (SD) age: massage 29 (3), relaxation 30 (2); range 23–35 USA: 46% Caucasian, 11% Hispanic, 38% African American and 4% other	Between 14 and 30 weeks gestation; gestational weeks Massage: 23.3 (4) PMR Relaxation: 23.6 (5)	Two groups: massage therapy by trained massage therapists; PMR relaxation therapy group at home alone; 20 min sessions twice a week for 5 weeks	STAI; Profile of Mood States Scale (POMS); CDC-D; pain VAS; The Sleep Disturbance Scale (Verran & Snyder-Halpern, 1988); urine: cortisol, catecholamines (norepinephrine, epinephrine, dopamine) and serotonin (5-HIAA)	Immediate effects (pre post session): Massage group: less STAI, POMS, less leg and back pain; relaxation group: less leg pain; Long-term effects (first session vs. last session): massage group: less sleep disturbance; less norepinephrine; more dopamine; PMR group: more dopamine	Fewer premature births in massage group	Not assessed	Fewer obstetric complications, fewer postnatal complications in massage group	Not reported
[32]	75 nulliparous pregnant women; 25 per group mean (SD) age: massage: 22.76 (3.85), guided imagery: 23.76 (3.74), control: 23.92 (4.41); Iran: Persians and Baloch	GA massage: 22.12 ± 0.93 GA-guided imagery: 22.20 (0.87) GA control: 22.12 (0.93)	Three groups: massage therapy (20 min once a week, for six weeks; Guided Imagery group (once a week by experimenter; every day video CD; Control group standard care	Pregnancy-related Anxiety Questionnaire—Revised [43]	Long-term effects (first session vs. last session): Massage and guided imagery (GI): sign. less anxiety compared to control group; no diff between massage and GI; Control group: sign. *more* anxiety post intervention	Not assessed	Not assessed	Not assessed	Only healthy women with low-risk pregnancy included
[33]	150 pregnant women, 75 per group; age mean (SD): 24.4 (4.35), Range: 19 to 33	Between 14 and 30 weeks GA; massage group 26.3 (3.77), control group 26.6 (3.81)	Two groups: Massage by confidante at home; 10–20 min twice/week over 5 week: Control group standard care	VAS pain; VAS sleep; Pregnancy-related Anxiety Questionnaire	Massage group less pregnancy discomforts: headache, backache, muscle cramp, sleep disturbance and anxiety; no diff. between groups in joint pain	Not assessed	Not assessed	Not assessed	Excluded were women who had high risk pregnancy or abnormal fetal condition
[34]	208 were screened for depression, 84 prenatally depressed women were analyzed; age: M = 26.6 years (Range 18–40); 38% Hispanic, 40% African American, and 12% Non-Hispanic White	Between 18 and 23 weeks GA	Three groups: Yoga (group sessions); professional massage therapy; standard prenatal care control group; 12 weeks twice weekly yoga or massage therapy sessions (20 min each)	SCID; Sociodemographic/Social Support Questionnaire; CES-D; STAI; STAXI; Pain VAS; Relationship Questionnaire	Long-term effects (first session vs. last session): Yoga group and massage group had greater decrease of depression, anxiety, back and leg pain and a greater increase on relationship scale than control group	Yoga and massage groups had greater gestational age and birthweight than the control group	Not assessed	Not assessed	Uncomplicated pregnancy
[35]	Number of participants unclear: 47 or 57 **depressed** (subclinical) pregnant women and their partners; between 18 and 40 years old (M = 27.9); 59% Hispanic, 32% Black and 9% Caucasian	2nd trimester	Two groups: Massage from partner; control group standard care; two 20 min massages per week; duration unclear: 12 or 16 weeks	SCID; Sociodemographic/Social Support Questionnaire; CES-D; STAI; STAXI; Pain VAS	Long-term effects (first session vs. last session): Massaged mothers: decreased leg pain and back pain, decreased depression, anxiety and anger, improved relationship with partner; fathers who massaged their partners versus the control group fathers: decreased depression and anxiety, improved relationship with partner	Not assessed	Not assessed	Not assessed	Healthy mothers with uncomplicated pregnancies
[36]	88 were screened, 61 randomized, 54 analyzed: pregnant women with **major depressive** disorder and a Hamilton Rating Scale for Depression score > 14; mean (SD) age: 33.3 (4.7); Caucasian 75%	Between 11 and 28 weeks GA; M = 20.0 (5.6)	Three groups: acupuncture specific for depression (ASD; *n* = 16), non-specific acupuncture (NASD; *n* = 19), massage (*n* = 19); 8 weeks; 12 sessions (25–30 min each)	Hamilton Rating Scale for Depression; BDI; SCID	Long-term effects (first session vs. last session): reduction in depressive symptoms in all three groups; no difference between groups	Not assessed	Not assessed	Not assessed	Not reported
[37]	183 depressed women recruited, 150 randomized, 141 began treatment; 33 discontinued treatment; mean (SD) age: ASD 32.4 (4.0); NASD 33,4 (5.0); Massage 32.8 (5.6)	GA week: ASD 19.8 (6.2); NASD 21.29 (5.4); Massage 21.06 (5.6)	Three groups: Acupuncture specific for depression (ASD; *n* = 49); Non-specific acupuncture (NASD; *n* = 44); Massage (*n* = 48); intervention 25 min, two times per week for the first 4 weeks and weekly for 4 more weeks;	Hamilton Rating Scale for Depression; BDI; SCID	Long-term effects (first session vs. last session): reduction in depressive symptoms in all three groups, highest decrease in ASD	Not assessed	Massage: 2% of women reported side effects (tiredness after treatment); Acupuncture: almost half of participants experienced mild or transient side effects (e.g., nausea, headache or sleep disturbance after treatment)	Ten unexpected/adverse events occurred: (1) premature delivery of twins with one neonatal demise and the surviving twin receiving prolonged neonatal intensive care (ASD); (2) pregnancy loss (NASD); (3) congenital defects among two neonates (one ASD, one **prenatal massage**); (4) hospitalization for esophageal spasms (**prenatal massage**); (5) hospitalization with dehydration and low amniotic fluid (NASD); 6) hospitalization for isolated atrial fibrillation (**prenatal massage**); (7) hospitalization because of premature contractions (**prenatal massage**); (8) preeclampsia (two in NASD). **The study investigators and the Data Safety and Monitoring Board classified all events as unrelated to treatment.**	Complications were no exclusion criteria, but 10 women dropped out due to pregnancy complications
[38]	320 were screened for depression; 112 pregnant women were diagnosed w depression and randomized; 48 were analyzed; mean (SD) age: PT: 23.71 (7.17), PT plus massage: 26.77 (5.19); 27% Hispanic, 68% Black and 5% White	Weeks GA: PT: 21.65 (4.44) PT plus massage: 19.98 (5.57)	Two groups: Inter-personal group psychotherapy (PT, *n* = 21); Inter-personal group psychotherapy plus massage (*n* = 27); PT 1 h per week; professional massage 20 min per week for 6 weeks	SCID; CES-D; STAI; STAXI; Relationship Questionnaire	PT plus massage: attended more sessions on average, and a greater percentage completed the 6-week program; Long-term effects (first session vs. last session): greater decrease in depression, depressed affect, somatic vegetative symptoms, anxiety and cortisol	No difference in birth weight or GA at birth	Not assessed	Not assessed	Not reported
[39]	113 assessed; 44 randomized; 27 women/partner dyads took part; Massage: *n* = 14 dyads, control: *n* = 13 dyads; women with self-reported mild-to-moderate anxiety (0–100 VAS scale) Age 19 to 41 years (mean age 29 years)	Between 28 and 32 weeks gestation	Two groups: Partner-delivered relaxation massage; control group: self-directed stress management training; at least one 20 min session per week till birth	DASS-21 subscale scores (anxiety, depression, stress) online questionnaire, every 4 weeks; Adverse events were reported weekly in online diary	Long-term effects (first vs. last day of study): Both programs decreased women’s symptoms of anxiety, depression and stress with no significant differences identified between the two groups	No difference between groups	Massage group: only two adverse events were reported: exacerbation of anxiety and tiredness. Control group: 12 participants reported adverse events, including sleeping problems (*n* = 4), pain (*n* = 3), exhaustion (*n* = 2), anxiety (*n* = 2)	No complications occurred	Only women with low-risk pregnancy included
[40]	93 screened, 62 met inclusion criteria, 52 took part; Mean (SD) age: 33.31 (4.01), range 24–43 years; Chinese	Mean 16 weeks GA	Two groups: massage (*n* = 24); control (*n* = 28); 70 min of aromatherapy massage with 2% lavender essential oil every other week (10 times in total) for 20 weeks; control group routine prenatal care	Salivary cortisol and immunoglobulin A (IgA) levels were collected before and after massage every 4 weeks (16, 20, 24, 30, 32, 36 weeks GA)	Immediate effects (pre/post session): Massage: lower cortisol and higher IgA levels at all six timepoints; no change in CO; Longitudinal: pre-test cortisol remained stable in massage group but increased successively in control group; pre-test IgA increased successively in massage group but not in control group	Not assessed	Not reported	Not reported	Healthy, low-risk, uncomplicated pregnancies

SCID: Structured Clinical Interview for DSM-IV Axis I Disorders; CES-D: Center for Epidemiological Studies-Depression Scale; BDI: Becks Depression Inventory; STAI: State Trait Anxiety Inventory; STAXI: State Anger Inventory; VAS: visual analogue scale.
